# Expired Patents

**Published:** 1907-03

**Authors:** 


### PATENTS.

### EXPIRED PATENTS.

January 28, 1907.

418108. Dental Apparatus. Samuel A. Milton. Clinton, Mo.

January 28, 1907.

420069. Dental Bracket. John Hood and Stephen H. Reynolds, Boston Mass.

420186. Dental Anodyne. Alfred Clark, Montpelier, Vt.

February 4, 1907.

420532. Dental-Plugger. Henry Craigie, San Francisco, Cal.

420653. Dental Anaesthetic. Robert A. Graham, Carrollton, Ohio.

420745. Dental Plugger. Edward M. Stroud, Pittston Pa.

420876. Rubber-Dam Clamp. Henry H. Johnson, and Sylvester G. Hill, London, England.

February, 11, 1907.

421116. Artificial Denture. John A. Throckmorton, Sidney, Ohio.

421250. Dental Forceps. Carter H. Dean, Norborn, Mo.

421328. Dental Engine. Carl H. Seeger, Manitowoc, Wis.

The above list of patterns furnished by Davis & Davis, solicitors of American and Foreign patents, Washington, D. C. and St. Paul Building New York City.

